# Reversal of Frailty and Improvement in Quality of Life Following Advanced Therapy Initiation in Patients with Inflammatory Bowel Disease: A Prospective Cohort Study

**DOI:** 10.3390/medicina62061192

**Published:** 2026-06-21

**Authors:** Mihaela Topala, Victor Ionescu, Monica Cojocaru, Razvan Iacob, Liliana Simona Gheorghe, Roxana Vadan, Cristian Gheorghe

**Affiliations:** 1 Doctoral School, Carol Davila University of Medicine and Pharmacy, 020021 Bucharest, Romania; 2 Center of Gastroenterology and Hepatology, Fundeni Clinical Institute, 022328 Bucharest, Romania; monicaluciacojocaru@yahoo.com (M.C.); raziacob@gmail.com (R.I.); drlgheorghe@gmail.com (L.S.G.); roxanavadan@yahoo.com (R.V.); drcgheorghe@gmail.com (C.G.); 3 Infection Control Unit, Bucharest Clinical Emergency Hospital, 014461 Bucharest, Romania; v.stefan@ymail.com; 4 Center for Excellence in Translational Medicine, Fundeni Clinical Institute, 022328 Bucharest, Romania; 5 Discipline of Gastroenterology and Hepatology, Department of Internal Medicine, Carol Davila University of Medicine and Pharmacy, 020021 Bucharest, Romania

**Keywords:** frailty, sarcopenia, quality of life, inflammatory bowel disease, Crohn’s disease, ulcerative colitis

## Abstract

*Background and Objectives*: In recent years, frailty has emerged as a prognostic factor in inflammatory bowel diseases (IBD), particularly among patients with active disease. However, evidence regarding its reversibility after treatment optimization remains limited. This study aimed to assess frailty in active IBD and determine whether frailty status improved after 6 months of clinical management and the achievement of clinical remission. *Materials and Methods*: This prospective, single-center, observational cohort study included adults with active IBD requiring escalation to advanced therapy who achieved clinical remission at the 6-month follow-up. Patients were evaluated at baseline and after 6 months using a modified Fried frailty phenotype. Quality of life was assessed using the Short Inflammatory Bowel Disease Questionnaire (SIBDQ), and depressive symptoms were assessed using the Center for Epidemiologic Studies Depression (CES-D) scale. Univariate and multivariate logistic regressions were utilized to identify independent factors associated with frailty improvement. *Results*: The analysis included 54 patients (61.1% male; 42.6% with Crohn’s disease). At baseline, 20.4% were classified as frail, 72.2% as pre-frail, and 7.4% as robust. Following 6 months of clinical management and the achievement of clinical remission, a 100% resolution of frailty was observed, with the robust cohort expanding to 42.6%. Significant improvements occurred across clinical parameters, including handgrip strength, 400 m walk times, and median SIBDQ scores (increasing from 4.4 to 5.9, *p* < 0.001) alongside a substantial decline in CES-D scores (*p* = 0.017). Multivariate logistic regression revealed that severe disease at baseline (aOR = 4.51, 95%CI: 1.26–16.18, *p* = 0.020), anti-TNF therapy initiation (aOR = 3.69, 95%CI: 1.04–13.18, *p* = 0.044), and higher baseline CES-D scores (aOR = 1.06, 95%CI: 1.00–1.13, *p* = 0.038) were independently associated with higher odds of frailty improvement. *Conclusions*: Among patients who achieved clinical remission, frailty and pre-frailty demonstrate substantial short-term improvement following advanced therapy. Functional and psychological recoveries are associated with successful control of baseline disease severity and systemic inflammation.

## 1. Introduction

Inflammatory bowel diseases (IBD), including ulcerative colitis (UC) and Crohn’s disease (CD), are characterized by flares of intestinal inflammation following a progressive and recurrent pattern, which produce systemic effects and carry a high risk of complications [1]. The course of the disease has a substantially negative impact on patients’ well-being, considering that less than half of all patients maintain remission under advanced treatments [2]. Chronic inflammation in conjunction with restrictions in oral intake, persistent diarrheal symptoms, and the impairment of digestive processes contribute to the development of nutritional deficiencies [3], and high rates of malnutrition and sarcopenia [4], along with a decreased quality of life (QoL) and high psychological distress [5].

Frailty represents a multidimensional syndrome characterized by decreased functional reserve of major organ systems and increased vulnerability to stressors, for which various assessment instruments have been proposed [6]. Fried et al. established one of the landmark definitions of frailty as a clinical syndrome encompassing at least three of the following components: unintentional weight loss, low grip strength, exhaustion, slow gait and low physical activity [7]. Although it was initially described in geriatric populations, frailty has emerged as a prognostic factor in chronic diseases and surgical settings [8,9,10,11,12].

In recent years, frailty has received increasing attention in IBD. Meta-analyses have estimated that frailty is frequent among IBD patients, with an 18% prevalence. Higher rates have been described in certain groups, such as patients with active disease, hospitalized patients, and elderly cohorts. Frailty is also linked to adverse outcomes related to the disease, being associated with higher risk of infections, an increased likelihood of post-operative morbidity, and higher odds of mortality [13]. However, frailty appears to be highly reversible. Kochar et al. reported that favorable response to anti-TNF (tumor necrosis factor) therapy initiation increased the odds of frailty improvement, with 85% of the frail patients ameliorating their frailty status [14]. Salvatori et al. reported similar results in a smaller, prospective cohort of 58 patients. Biologic therapy increased the odds of frailty improvement by almost 22 times [15].

However, prospective data remain limited, particularly in younger adults, and few studies have combined frailty assessment with standardized physical-performance measures and patient-reported outcomes. Therefore, we aimed to assess frailty in hospitalized patients with active IBD and determine whether frailty status improved after 6 months of clinical management and the achievement of clinical remission. Additionally, we explored factors associated with frailty improvement and its relationship with QoL.

## 2. Materials and Methods

### 2.1. Data and Patient Population

This prospective, single-center, observational cohort study was conducted at Fundeni Clinical Institute, a tertiary academic referral center in Bucharest, Romania. The study protocol was approved by the Ethics Committee of Fundeni Clinical Institute (approval No. 46272, 8 September 2020) and conducted in accordance with the principles of the Declaration of Helsinki, as revised in 2013. Written informed consent was obtained from all participants before enrollment. Ethnicity was not systematically recorded in the study database.

Eligible participants were adults with a histopathologically confirmed diagnosis of UC, CD, or IBD-unclassified, who were hospitalized in the Department of Gastroenterology between January 2021 and March 2022, and between October 2023 and August 2025. The present analysis focused on the longitudinal evaluation of frailty and related clinical outcomes in this cohort. Patients were included if they had active disease requiring escalation to biological therapy or small-molecules. Patients were assessed at baseline and were eligible for the 6-month reassessment only if they had achieved clinical remission. At the follow-up, the examination protocol was repeated using the same clinical, frailty, functional, and patient-reported assessments. Patients with persistent clinically active disease or those requiring a change in advanced therapy were not included in the longitudinal analysis. Patients were also excluded if they were younger than 18 years, pregnant or lactating, unable or unwilling to provide informed consent, or had major comorbidities likely to affect physical or psychological performance. These comorbidities included heart failure, ischemic heart disease, chronic kidney disease, diabetes mellitus, chronic liver disease, chronic obstructive pulmonary disease, hematologic diseases, neurological disorders, malignancy, and severe psychiatric conditions.

The Crohn’s Disease Activity Index (CDAI) [16] was used to assess severity in CD patients—clinical remission was indicated by a score < 150 points, mildly active disease between 151 and 219, moderately active between 220 and 450, and severely active with a score > 450. The full Mayo score [17] evaluated UC severity at baseline as follows: clinical remission for 0–1 point, mild disease for 2–5, moderate disease for 6–9, and severe disease for 10–12. At the 6-month follow-up, clinical remission was defined as a CDAI score < 150 for CD, and a partial Mayo score ≤ 1 for UC. Endoscopic reassessment and fecal calprotectin measurements were not available for all patients, and, therefore, were not required to define remission at follow-up.

The National Health and Nutrition Examination Survey Anthropometry Procedures Manual (NHANES) recommendations were followed for the anthropometric measurements. Each measurement was repeated three times, and the mean value was used for analysis. Frailty was assessed using a modified version of the Fried frailty phenotype, comprising five domains: unintentional weight loss, exhaustion, weakness, slowness, and low physical activity [7]. Unintentional weight loss was defined as a loss of at least 4.5 kg or at least 5% of body weight during the previous year. Exhaustion was assessed using two items from the Center for Epidemiologic Studies Depression Scale (CES-D): “I felt that everything I did was an effort” and “I could not get going”. Weakness was defined as handgrip strength (HGS) below the 20th percentile for age and sex, using the normative reference data presented as means and standard deviations from the Jamar^®^ manual, based on Mathiowetz et al. [18]. The 20th percentile was estimated assuming a normal distribution, using the formula: mean − 0.842 × standard deviation. Slowness was defined as a gait speed below 0.8 m/s during the 4.5 m walk test [19]. Low physical activity was defined as the “Low” category of the International Physical Activity Questionnaire (IPAQ). The phenotype was considered modified because unintentional weight loss and exhaustion followed the original Fried definitions, whereas weakness, slowness, and low physical activity were operationalized using age- and sex-specific Mathiowetz HGS thresholds, a gait speed < 0.8 m/s during the 4.5 m walk test, and the IPAQ “Low” physical category, respectively. Participants meeting three or more criteria were classified as frail, those meeting one or two criteria as pre-frail, and those meeting none as robust, in accordance with the original Fried classification.

HGS was measured using a Jamar^®^ hydraulic dynamometer (Lafayette Instrument Company, Lafayette, Indiana, USA), according to the American Society of Hand Therapists recommendations [20]. Participants were seated with both feet on the floor, the elbow flexed at 90°, and the wrist in slight extension. The dynamometer was positioned so that the proximal interphalangeal joint of the ring finger formed a 90° angle, with the thumb and fingers aligned on the same side of the device. Muscle strength was further assessed using the five-repetition chair stand test. Physical performance was evaluated using the 400 m walk test and gait speed during the 4.5 m walk. All measurements except the 400 m walk test were performed three times, and the mean value was used for analysis. Mid-arm muscle circumference (MAMC) was calculated using the following formula: mid-upper arm circumference (MUAC) − π × triceps skinfold thickness (TSF). Body mass index (BMI) was calculated as weight in kilograms divided by height in meters squared.

QoL was assessed using the Short Inflammatory Bowel Disease Questionnaire (SIBDQ), which encompasses 10 questions covering four domains (Bowel, Emotional, Systemic, Social); lower scores indicate poor QoL [21]. The CES-D, a 20-item self-report questionnaire, was used to screen for depressive symptoms, with higher scores indicating an increased risk of depression [22].

### 2.2. Statistical Analysis

The distribution of continuous variables was assessed for normality using the Shapiro–Wilk test. Continuous variables were summarized as medians (interquartile ranges, IQRs) or means ± standard deviations (SDs), based on their distribution. Categorical variables were reported as frequencies and percentages. HGS Z-scores were also calculated using normative reference data. To evaluate the baseline differences among the frailty categories (frail, pre-frail, and robust), the Kruskal–Wallis test was used for continuous variables, and the Chi-square test or Fisher’s exact test was used for categorical data. Longitudinal changes across the three frailty categories were analyzed with the Exact Marginal Homogeneity test. Due to the complete absence of a frail cohort at 6 months (zero variance), longitudinal McNemar testing was omitted, and the results were evaluated descriptively. For paired comparisons between the baseline and 6-month follow-up, the distribution of within-patient differences was assessed. For parametric data, the paired t-test was performed; for non-parametric continuous variables, the Wilcoxon signed-rank test or the Sign test was used, depending on the symmetry of the parameter. The mathematical assumption of symmetry around the median was assessed by visually inspecting histograms and boxplots. Analyses were based on available complete observations for each assessment, and no missing values were imputed. Four patients were unable to complete the 400 m walk test at baseline but completed it at follow-up; therefore, the paired analysis of 400 m walk completion time included the 50 patients with numerical results at both time points.

Frailty improvement was defined as a transition to a more favorable frailty category between the baseline and the 6-month follow-up, including frail-to-pre-frail, frail-to-robust, and pre-frail-to-robust transitions. These transitions were pooled as the binary outcome “improvement.” Patients who remained in the same category or transitioned to a less favorable category were classified as having no improvement. Patients who were robust at baseline were classified as having no improvement because no more favorable category was available.

To identify factors associated with frailty improvement, logistic regression analyses were performed using complete cases for all included predictor variables. Variables with a *p* value < 0.100 in univariate logistic regression were entered into a multivariate logistic regression model. Given the modest cohort size, the multivariate model was restricted to a maximum of three parameters to reduce the risk of overfitting and improve estimate stability. Odds ratios (ORs) and 95% confidence intervals (CIs) were computed to quantify the strength of these associations. The model’s goodness-of-fit was assessed using the Hosmer–Lemeshow test and Nagelkerke R Square. Associations between changes in clinical parameters and QoL were assessed using Spearman’s rank correlation coefficient (*r_s_*). Differences in the magnitude of QoL improvement across different advanced therapy classes were assessed using the Mann–Whitney U test and Kruskal–Wallis test. All tests were two-sided, and a *p* value < 0.05 was considered statistically significant. All analyses were performed using IBM^®^ SPSS^®^ Statistics version 23.0.

## 3. Results

### 3.1. Baseline Characteristics

Seventy-five patients were initially enrolled (60.0% males; 58.7% with UC). Of these, five (6.7%) declined the 6-month reassessment, four (5.3%) were lost to follow-up, four (5.3%) were excluded because of persistent clinically active disease or a switch to another advanced therapy, four (5.3%) continued the follow-up at a regional hospital, three (4.0%) underwent surgical interventions for IBD-related complications, and one (1.3%) discontinued treatment on his own initiative. The final cohort comprised 54 patients with active IBD at baseline who were in clinical remission at the 6-month assessment. Of these, 33 (61.1%) were male and 23 (42.6%) were diagnosed with CD. The median age was 35.5 years, and the median disease duration was 5 years. Based on the Fried-derived frailty phenotype, 11 (20.4%) patients were classified as frail, 39 (72.2%) as pre-frail, and only 4 (7.4%) as robust. Frail patients had significantly higher baseline disease activity, with a median CDAI score of 425 compared with 234 among the pre-frail, and 152 among the robust patients (*p* = 0.013). Frailty was also associated with greater weight loss (median, 12 kg in the frail group vs. 6 kg in the pre-frail group, and 1 kg in the robust group; *p* = 0.001). No significant differences were observed across the frailty categories at baseline regarding age, sex, disease duration, IBD phenotype, presence of extraintestinal manifestations, or current treatment. Detailed baseline characteristics are presented in Table 1.

### 3.2. Follow-Up Assessment

All 54 patients completed the 6-month reassessment, and significant improvements were observed in several clinical and functional measures, as shown in Table 2. At baseline, four patients were unable to complete the 400 m walk test: one because of an urgent need for a bowel movement and three because of weakness. All four patients completed the test at the 6-month follow-up. Consequently, the paired comparison of 400 m walk completion times was restricted to the 50 patients with complete numerical measurements at both assessments.

Following the 6-month clinical management period, all 11 patients who were frail at baseline transitioned to either pre-frail or robust status; thus, no patient remained frail at follow-up. During follow-up, 12 patients required dose escalation of the initiated advanced therapy and maintained clinical remission under the optimized regimen. Five patients required corticosteroid therapy during follow-up but achieved clinical remission and discontinued corticosteroids before the 6-month reassessment. Patients treated with infliximab also received concomitant azathioprine to reduce the risk of immunogenicity. Nine patients (16.7%) developed infections: six (11.1%) had *Clostridioides difficile* infection, two (3.7%) had other types of infectious colitis, and one (1.9%) had a respiratory infection. The infection rate did not differ significantly according to the baseline frailty category (*p* = 0.706). Inflammatory biomarkers improved significantly: median C-reactive protein decreased from 15.0 to 1.9 mg/L (*p* < 0.001) and median thrombocytes dropped from 356.5 to 304.0 × 10^3^/µL (*p* < 0.001). Muscle strength, anthropometric measures, and physical performance also improved, with mean HGS increasing from 33.7 ± 11.7 to 36.6 ± 12.8 kgf (*p* < 0.001), median MAMC increasing from 19.7 to 20.5 cm (*p* = 0.019), and median 400 m walk completion time decreasing from 302.0 to 286.5 s (*p* < 0.001). Furthermore, health-related quality of life optimized significantly, as demonstrated by an increase in total SIBDQ scores from 4.4 to 5.9 (*p* < 0.001) alongside significant decrease in CES-D depression scores (9.0 to 6.0, *p* = 0.017).

Over 6 months, 27 (50.0%) patients experienced a frailty category transition (Table 3). Of these, 26 patients (48.1%) improved by at least one frailty category, while one patient (1.9%) transitioned from robust to pre-frail. The remaining 27 patients (50.0%) remained in the same frailty category. The overall distribution of frailty categories improved significantly (Exact Marginal Homogeneity test, *p* < 0.001), driving a reduction in the sample’s average frailty status metric from 1.13 ± 0.52 at baseline down to 0.57 ± 0.50 at the 6-month follow-up evaluation. All 11 patients who were frail at baseline improved: six patients (54.5%) successfully improved to a pre-frail state, while five patients (45.5%) achieved a full recovery to a robust phenotype. Among the 39 individuals starting in a pre-frail state, 24 (61.5%) remained stable, while 15 (38.5%) transitioned to a robust classification. Of the four patients who were robust at baseline, three (75.0%) remained robust, and only one (25.0%) became pre-frail. In summary, these individualized shifts drove a substantial expansion of the overall robust cohort from 7.4% up to 42.6% of the entire sample population.

### 3.3. Predictive Factors for Frailty Improvement

Univariate logistic regression showed that severe disease at baseline was significantly associated with higher odds of frailty improvement at 6 months (OR = 4.09, 95% CI: 1.29–13.00; *p* = 0.017). A higher baseline CES-D score was also associated with frailty improvement, although the effect was small (OR = 1.05, 95% CI: 1.00–1.11; *p* = 0.047). Anti-TNF initiation showed a borderline association with higher odds of frailty improvement (OR = 2.92, 95% CI: 0.96–8.84; *p* = 0.058). Vedolizumab initiation showed a nonsignificant trend toward lower odds of improvement (OR = 0.28, 95% CI: 0.07–1.16; *p* = 0.079). No other demographic, disease-related, treatment-related, inflammatory, nutritional, or patient-reported variables were significantly associated with frailty improvement.

In the exploratory multivariate logistic regression model, severe disease at baseline, anti-TNF initiation, and the baseline CES-D score were independently associated with frailty improvement (Table 4). Severe disease (aOR = 4.51, 95% CI: 1.26–16.18; *p* = 0.020) and anti-TNF initiation (aOR = 3.69, 95% CI: 1.04–13.18; *p* = 0.044) were both associated with higher odds of frailty improvement. A higher baseline CES-D score was also associated with improvement, although the effect was modest (aOR = 1.06, 95% CI: 1.00–1.13; *p* = 0.038).

### 3.4. Quality of Life and Psychological Outcomes

QoL, as measured by the SIBDQ, showed a profound improvement from a median of 4.4 at baseline to 5.9 at follow-up (*p* < 0.001). The most significant gains were noted in the Social domain (median increase from 4.5 to 7.0) and the Systemic domain (4.0 to 5.5). Concurrently, psychological distress scores on the CES-D improved from a median of 9.0 to 6.0 (*p* = 0.017).

The improvement in QoL was significantly associated with both physical and psychological recovery. Spearman correlation analysis revealed that the increase in total SIBDQ scores was positively correlated with gains in HGS (*r_s_* = 0.329, *p* = 0.017). Furthermore, a strong inverse correlation was observed between changes in CES-D scores and SIBDQ scores (*r_s_* = −0.483, *p* < 0.001). While improvements in the 400 m walk test showed a trend toward association with better SIBDQ scores (*r_s_* = −0.263, *p* = 0.074), no significant correlations were found between QoL changes and MAMC (*r_s_* = 0.052, *p* = 0.716) or physical activity levels assessed through IPAQ (*r_s_* = 0.109, *p* = 0.438).

To assess whether the therapy class influenced QoL recovery, subgroup analyses were performed. There were no statistically significant differences in the improvement of SIBDQ scores when comparing patients treated with anti-TNF agents (*p* = 0.550), vedolizumab (*p* = 0.617), ustekinumab (*p* = 0.209), or small molecules (*p* = 0.935). Furthermore, the overall comparison across all advanced therapy classes was also nonsignificant (*p* = 0.630).

## 4. Discussions

In this cohort of patients with active IBD, frailty and pre-frailty were highly prevalent at baseline but improved substantially after clinical remission was achieved. At 6 months, no patient remained frail, and most participants either improved or remained stable. These changes were accompanied by improvements in inflammatory biomarkers, physical performance, nutritional parameters, and health-related QoL, suggesting that frailty in active IBD is closely related to disease activity and overall clinical burden. Our results showed that severe disease at baseline and anti-TNF initiation were independently associated with frailty improvement. Although a higher baseline CES-D score was also linked to improvement in frailty, the effect size was modest.

Studies have reported different rates of frailty categories depending on the inpatient/outpatient status, disease activity, age distribution, and comorbidities. Moreover, the results might have varied according to the frailty assessment instrument that was used. Scores that are based solely on comorbidities or administrative codes, such as International Classification of Diseases (ICD) diagnoses, may not reflect the clinical reality accurately. Thus, claims-based indices and phenotype-based approaches capture different dimensions of frailty. Previous studies have reported frailty rates of 13.0–21.7% and pre-frailty rates of 28.0–59.4% in patients with IBD [23,24,25,26]. The cohort in our study presented higher rates of pre-frailty (72.2%); however, this result aligns with the disease severity since all patients had active disease at baseline, requiring the initiation of advanced therapy.

Frailty is recognized as a predictor of negative outcomes in the elderly population, and screening recommendations have been implemented in the geriatric guidelines [27]. Regarding the elderly IBD population, the role of frailty in optimizing management and treatment strategies has already been acknowledged in topic reviews from the European Crohn’s and Colitis and the American Gastroenterology Association [28,29]. One important aspect in frailty identification is linked to the possibility of frailty reversal. Frailty represents a dynamic condition in which shifting between stages is possible, both in positive and negative directions. Physical activity and nutritional interventions can improve frailty in the general population [30]; however, in IBD, frailty may be strongly influenced by disease activity and severity.

Two studies have evaluated frailty improvement after IBD treatment. In a retrospective cohort of 1210 patients with IBD from a large healthcare system, Kochar et al. showed that 85% of the frail individuals presented a favorable change in their frail phenotype within a year from anti-TNF initiation. The cohort was relatively young, with a median age of 30 years, and there was no restriction to a specific inpatient or outpatient setting. Frailty was indirectly assessed using a claims-based frailty index calculated during the year before and after treatment initiation. Clinical response to IBD therapy was independently associated with frailty improvement, while pre-treatment frailty and prior hospital admission were predictive for frailty persistence after treatment initiation. Moreover, patients with higher scores of pre-treatment frailty experienced better improvement, including those older than 50 years at the time of therapy initiation [14]. Salvatori et al. prospectively followed 58 tertiary-center outpatients who were all frail at baseline, with a median age of 54 years. Frailty was assessed using the Fried phenotype. After a median of 8 months of clinical management, 81% of patients improved their frailty status. The presence of extraintestinal manifestations and persistently active disease were associated with the maintenance of a frail status, while biologic therapy was a strong predictor of frailty improvement [15].

Our cohort consisted of hospitalized patients with active IBD requiring treatment escalation. The median age was 35.5 years, which was higher than in the Kochar cohort but lower than in the Salvatori cohort. We also included patients across the full frailty spectrum at baseline and prospectively reassessed them at a fixed 6-month interval using a Fried-derived phenotype together with standardized measures of muscle strength, physical performance, nutritional status, QoL, and depressive symptoms. Severe disease at baseline, anti-TNF initiation, and higher baseline CES-D scores were associated with frailty improvement. These findings suggest that patients with a greater baseline disease burden or symptom burden may be more likely to experience improvements in frailty, possibly reflecting a greater potential for functional recovery after effective disease control. The CES-D association may similarly reflect a greater baseline psychological and symptom burden and, therefore, a greater potential for improvement after remission. However, the effect size was modest and the confidence interval was close to unity; this finding should be regarded as exploratory and should not be interpreted as evidence that depressive symptoms promote frailty improvement. The association with anti-TNF initiation may reflect that treatment-related inflammatory control contributed to frailty improvement. However, given the modest sample size and the wide confidence intervals, particularly for anti-TNF initiation, the multivariate findings should be regarded as exploratory rather than confirmatory. No patient remained frail at follow-up, but the longitudinal analysis included only patients who achieved clinical remission. Therefore, the complete resolution of the frail category should be interpreted in the context of this selected responder population.

Health-related QoL is commonly impaired in IBD, particularly in patients with active or severe disease, but may improve over time [31]. Sarcopenia and malnutrition are associated with lower scores of QoL, whereas physical activity has proved to have a beneficial effect [32,33]. In an elderly IBD cohort, deficits across geriatric assessment domains, including mental health, somatic status, activities of daily living, and physical capacity, were independently associated with lower QoL [34]. Frailty has also been associated with subsequent deterioration in QoL and functional status [35]. In non-geriatric patients, the relation between QoL and frailty has been incompletely investigated. However, sleep impairment and depression appear to be independent factors associated with frailty [26,36]. In our cohort, physical recovery was accompanied by a substantial improvement in QoL. Improvements occurred across all SIBDQ domains, but they were more predominant in the Systemic and Emotional domains. The concurrent decrease in CES-D scores suggests that reduced disease burden may be accompanied by fewer depressive symptoms. Although anti-TNF initiation was associated with frailty improvement, QoL improved irrespective of the advanced therapy class. These findings support a multidimensional assessment of outcomes in IBD, but do not establish that psychological improvement was caused solely by the control of inflammation.

Our study has several strengths. First, it focused on frailty among hospitalized patients with active IBD, a clinically relevant topic that has received limited attention to date. Second, its longitudinal design enabled reassessment of frailty after 6 months of clinical management, highlighting the possibility of frailty improvement over time, with appropriate intervention in this population. Third, the study incorporated a multidimensional evaluation, including clinical activity, inflammatory markers, nutritional and anthropometric measures, physical performance, muscle strength, QoL, and depressive symptoms. This approach provided a comprehensive assessment of frailty and its related outcomes.

Several limitations should be acknowledged. The modest sample size limited the statistical power and the precision of the regression estimates. Because the study was conducted at a single tertiary academic referral center in Romania, the findings may not be generalizable to broader IBD populations, community settings, or other healthcare systems. Only patients who achieved remission and completed the follow-up were analyzed, which might limit generalizability to patients with refractory or persistently active disease. This selection might have overestimated the magnitude of frailty improvement because patients with persistently active disease, treatment failure, or therapy switching, who might have remained frail, were not represented. These findings should not be generalized to all patients initiating advanced therapy. Information on nutritional supplementation, psychological support, physical rehabilitation, and other complementary interventions was not systematically collected; therefore, their potential contribution to frailty improvement could not be assessed. Remission at follow-up was defined using clinical indices only, because endoscopic reassessment or fecal calprotectin measurements were not available for all patients. Therefore, residual inflammatory activity could not be excluded. The follow-up was limited to 6 months, and studies with longer observation periods are needed to establish whether frailty improvement is sustained. Finally, although age- and sex-specific HGS reference values were used, the frailty-component thresholds have not been specifically validated in younger patients with IBD.

These findings require confirmation in larger, multicenter cohorts with longer follow-up periods. Future studies should evaluate whether frailty improvement is sustained and whether recurrent disease activity, treatment discontinuation, hospitalization, or surgery influences long-term frailty trajectories. Interventional studies combining optimized advanced therapy with nutritional support, structured physical exercise, and psychological interventions may help identify the components that most effectively promote improvement. The development and validation of IBD-specific frailty assessment tools would also be valuable, particularly for younger patients in whom traditional geriatric frailty instruments may not fully capture disease-related vulnerability.

## 5. Conclusions

Among patients with active inflammatory bowel disease who achieved clinical remission, frailty and pre-frailty improved substantially after 6 months of clinical management. Frailty improvement was accompanied by reductions in inflammatory markers and improvements in physical performance, nutritional parameters, depressive symptoms, and quality of life. In an exploratory multivariate analysis, severe disease at baseline and anti-TNF initiation were associated with higher odds of frailty improvement, suggesting that effective control of the inflammatory burden may contribute to functional recovery. Frailty in active inflammatory bowel disease appears to be a dynamic and potentially reversible condition. However, larger and more representative studies are needed before frailty assessment can be recommended for routine multidisciplinary care in inflammatory bowel disease.

## Figures and Tables

**Table 1:** Baseline characteristics across frailty categories of the included patients.

Variable	Total Cohort (n = 54)	Frail (n = 11)	Pre-Frail (n = 39)	Robust (n = 4)	*p* Value
Age (years), median (IQR)	35.5 (25.0–44.0)	32.0 (26.0–35.0)	36.0 (27.0–44.0)	41.0 (38.2–46.7)	0.124
Male sex, n (%)	33 (61.1%)	5 (45.5%)	26 (66.7%)	2 (50.0%)	0.449
Disease duration (years), median (IQR)	5.0 (1.0–10.0)	1.0 (0.0–6.0)	5.5 (1.0–10.0)	9.0 (4.0–12.5)	0.233
Crohn’s disease, N (%)	23 (42.6%)	4 (36.4%)	16 (41.0%)	3 (75.0%)	0.463
Age at diagnosis					
≤16 years, n/N (%)	2/23 (8.7%)	1/4 (25.0%)	1/16 (6.3%)	0/3 (0.0%)	0.526
17–40 years, n/N (%)	18/23 (78.3%)	3/4 (75.0%)	12/16 (75.0%)	3/3 (100.0%)	1.000
>40 years, n/N (%)	3/23 (13.0%)	0/4 (0.0%)	3/16 (18.8%)	0/3 (0.0%)	1.000
Disease location					
Terminal ileum, n/N (%)	4/23 (17.4%)	1/4 (25.0%)	2/16 (12.5%)	1/3 (33.3%)	0.352
Colonic, n/N (%)	6/23 (26.1%)	2/4 (50.0%)	3/16 (18.8%)	1/3 (33.3%)	0.373
Ileocolonic, n/N (%)	13/23 (56.5%)	1/4 (25.0%)	11/16 (68.8%)	1/3 (33.3%)	0.288
Disease behavior					
Inflammatory, n/N (%)	10/23 (43.5%)	0/4 (0.0%)	7/16 (43.8%)	3/3 (100.0%)	0.036
Stricturing, n/N (%)	8/23 (39.1%)	2/4 (50.0%)	6/16 (37.5%)	0/3 (0.0%)	0.537
Penetrating, n/N (%)	5/23 (21.7%)	2/4 (50.0%)	3/16 (18.8%)	0/3 (0.0%)	0.292
Perianal lesions, n/N (%)	7/23 (30.4%)	2/4 (50.0%)	4/16 (25.0%)	1/3 (33.3%)	0.786
Ulcerative colitis, N (%)	31 (57.4%)	7 (63.6%)	23 (59.0%)	1 (25.0%)	0.463
Left-sided, n/N (%)	11/31 (35.5%)	2/7 (28.6%)	8/23 (34.8%)	1/1 (100.0%)	0.595
Extensive, n/N (%)	20/31 (64.5%)	5/7 (71.4%)	15/23 (65.2%)	0/1 (0.0%)	0.595
Extraintestinal manifestations, n (%)	5 (9.3%)	1 (9.1%)	4 (10.3%)	0 (0.0%)	1.000
Prior IBD-related surgery, n (%)	6 (11.1%)	3 (27.3%)	3 (7.7%)	0 (0.0%)	0.146
Disease severity, n (%)					
Severe disease, n (%)	22 (40.7%)	7 (63.6%)	14 (35.9%)	1 (25.0%)	0.216
CDAI score (23 patients)	228.0 (167.0–397.0)	425.0 (265.0–462.0)	234.0 (181.0–303.7)	152.0 (151.0–154.0)	0.013
Mild CD, n/N (%)	8/23 (34.8%)	0/4 (0.0%)	5/16 (31.3%)	3/3 (100.0%)	0.025
Moderate CD, n/N (%)	10/23 (43.5%)	2/4 (50.0%)	8/16 (50.0%)	0/3 (0.0%)	0.355
Severe CD, n/N (%)	5/23 (21.7%)	2/4 (50.0%)	3/16 (18.8%)	0/3 (0.0%)	0.292
Full Mayo score (31 patients)	11.0 (9.0–11.0)	11.0 (10.0–12.0)	10.0 (8.0–11.0)	11 *	0.224
Moderate UC, n/N (%)	14/31 (45.2%)	2/7 (28.6%)	12/23 (52.2%)	0/1 (0.0%)	0.412
Severe UC, n/N (%)	17/31 (54.8%)	5/7 (71.4%)	11/23 (47.8%)	1/1 (100.0%)	0.412
Current treatment at baseline					
5-Aminosalicylates, n (%)	26 (48.1%)	5 (45.5%)	19 (48.7%)	2 (50.0%)	1.000
Corticosteroids, n (%)	29 (53.7%)	8 (72.7%)	19 (48.7%)	2 (50.0%)	0.343
Immunomodulators, n (%)	10 (18.5%)	0 (0.0%)	9 (23.1%)	1 (25.0%)	0.178
Advanced therapies, n (%)	16 (29.6%)	3 (27.3%)	13 (33.3%)	0 (0.0%)	0.609
Advanced therapy initiated					
Anti-TNF, n (%)	28 (51.9%)	8 (72.7%)	18 (46.2%)	2 (50.0%)	0.296
Vedolizumab, n (%)	12 (22.2%)	1 (9.1%)	11 (28.2%)	0 (0.0%)	0.283
Ustekinumab, n (%)	10 (18.5%)	1 (9.1%)	7 (17.9%)	2 (50.0%)	0.221
Small molecules, n (%)	4 (7.4%)	1 (9.1%)	3 (7.7%)	0 (0.0%)	1.000
Weight loss (kg), median (IQR)	6.7 (4.0–12.0)	12.0 (10.0–15.0)	6.0 (4.0–11.0)	1.0 (0.0–2.7)	0.001
Period of weight loss (months), median (IQR)	1.0 (0.8–5.0)	4.0 (1.5–6.0)	3.0 (2.0–4.5)	0.7 (0.5–1.0)	0.081

Abbreviations: CD—Crohn’s disease; CDAI—Crohn’s Disease Activity Index; IBD—inflammatory bowel disease; IQR—interquartile range; kg—kilograms; n—number; TNF—tumor necrosis factor; UC—ulcerative colitis. Percentages for Crohn’s disease-specific variables were calculated among patients with Crohn’s disease. Percentages for ulcerative colitis-specific variables were calculated among patients with ulcerative colitis. * Exact value reported for one patient.

**Table 2:** Baseline vs. 6-month follow-up characteristics of the included patients.

Variable	Baseline	6-Month Follow-Up	*p*
BMI (kg/m^2^), mean ± SD	21.6 ± 3.6	23.3 ± 3.4	<0.001
Laboratory tests			
C reactive protein (mg/L), median (IQR)	15.0 (5.0–52.5)	1.9 (0.8–4.7)	<0.001
Fibrinogen (mg/dL), mean ± SD	479.1 ± 135.6	347.1 ± 81.0	<0.001
Hemoglobin (g/dL), mean ± SD	12.8 ± 2.1	13.6 ± 1.8	0.001
White blood cells (×10^3^/uL), mean ± SD	9.5 ± 3.2	7.2 ± 2.0	<0.001
Thrombocytes (×10^3^/μL), median (IQR)	356.5 (288.5–445.8)	304.0 (257.5–343.0)	<0.001
Albumin (g/dL), mean ± SD	4.2 ± 0.7	4.6 ± 0.5	<0.001
Waist (cm), mean ± SD	82.0 ± 10.9	85.3 ± 11.3	<0.001
Mid-arm muscle circumference (cm), median (IQR)	19.7 (16.6–22.2)	20.5 (17.2–22.7)	0.019
Handgrip strength (kgf), mean ± SD	33.7 ± 11.7	36.6 ± 12.8	<0.001
HGS Z-score, mean ± SD	−1.3 ± 0.9	−1.0 ± 0.9	0.001
Chair stand test (seconds), mean ± SD	12.4 ± 3.7	11.8 ± 2.6	0.158
4.5 m test (seconds), median (IQR)	3.8 (3.3–4.3)	3.5 (3.2–3.7)	0.001
400 m test (seconds), median (IQR), n = 50 *	302.0 (282.3–330.3)	286.5 (271.3–304.3)	<0.001
SARC-F (points), median (IQR)	0.0 (0.0–2.0)	0.0 (0.0–1.0)	0.263
IPAQ (MET-min/Wk), median (IQR)	3360.0 (1305.0–6469.5)	4203.0 (2388.4–9164.5)	0.031
CES-D (points), median (IQR)	9.0 (3.0–20.0)	6.0 (3.0–9.0)	0.017
SIBDQ (points), median (IQR)	4.4 (3.3–5.6)	5.9 (5.2–6.5)	<0.001
Systemic (points), median (IQR)	4.0 (3.0–5.5)	5.5 (5.0–6.0)	<0.001
Social (points), median (IQR)	4.5 (2.5–6.5)	7.0 (5.5–7.0)	<0.001
Bowel (points), median (IQR)	4.7 (3.3–5.8)	6.0 (5.2–6.7)	<0.001
Emotional (points), median (IQR)	4.7 (3.5–6.0)	5.7 (5.0–6.5)	<0.001
Frailty Phenotype Status			
Robust, n (%)	4 (7.4%)	23 (42.6%)	<0.001
Pre-frail, n (%)	39 (72.2%)	31 (57.4%)	
Frail, n (%)	11 (20.4%)	0 (0.0%)	

Abbreviations: CES-D—Center for Epidemiologic Studies Depression; cm—centimeters; dl—deciliter; g—gram; HGS—handgrip strength; IPAQ—International Physical Activity Questionnaire; IQR—interquartile range; kg—kilogram; kgf—kilogram-force; l—liter; m—meter; mg—milligram; Met-min—Metabolic Equivalent of Task minutes; SIBDQ—Short Inflammatory Bowel Disease Questionnaire; SD—standard deviation; μL—microliter; Wk—week; * Four patients were unable to complete the 400 m walk test at baseline but completed it at follow-up. The paired comparison of completion times therefore included 50 patients. No values were imputed.

**Table 3:** Shift table of frailty status from baseline to 6-month follow-up.

	6-Month Follow-Up Status			
Baseline Status	Robust (n = 23)	Pre-Frail (n = 31)	Frail (n = 0)	Total (n = 54)
Robust (n = 4)	3 (75.0%)	1 (25.0%)	0	4 (100.0%)
Pre-frail (n = 39)	15 (38.5%)	24 (61.5%)	0	39 (100.0%)
Frail (n = 11)	5 (45.5%)	6 (54.5%)	0	11 (100.0%)
Total (n = 54)	23 (42.6%)	31 (57.4%)	0	54 (100.0%)

Abbreviations: n—number.

**Table 4:** Results of univariate and multivariate logistic regression analyses of factors associated with frailty improvement in patients with inflammatory bowel disease.

	Univariate Analysis		Multivariate Analysis	
Predictor	Odds Ratio (95% Confidence Interval)	*p*	Odds Ratio (95% Confidence Interval)	*p*
Age	1.01 (0.97–1.06)	0.575		
Male sex	1.95 (0.64–5.95)	0.241		
Disease duration	1.00 (0.93–1.09)	0.847		
Crohn’s disease	0.72 (0.24–2.13)	0.555		
Body mass index	0.95 (0.81–1.10)	0.466		
Severe disease	4.09 (1.29–13.00)	0.017	4.51 (1.26–16.18)	0.020
C-reactive protein	1.01 (1.00–1.03)	0.077		
Albumin level	0.46 (0.20–1.08)	0.075		
Prior advanced therapy	0.78 (0.24–2.52)	0.675		
Prior corticosteroids	1.85 (0.62–5.46)	0.268		
Anti-TNF initiation	2.92(0.96–8.84)	0.058	3.69 (1.04–13.18)	0.044
Vedolizumab initiation	0.28 (0.07–1.16)	0.079		
Ustekinumab initiation	0.67 (0.17–2.69)	0.569		
Small-molecule initiation	1.08 (0.14–8.30)	0.939		
No infections during follow-up	2.09 (0.47–9.41)	0.336		
Surgery history	6.43 (0.70–59.28)	0.101		
Extraintestinal manifestations	0.24 (0.06–2.30)	0.216		
CES-D score	1.05 (1.00–1.11)	0.047	1.06 (1.00–1.13)	0.038

Abbreviations: CES-D—Center for Epidemiologic Studies Depression; TNF—tumor necrosis factor.

## Data Availability

Data used for the statistical analyses can be shared on request to the corresponding author.

## Abbreviations

The following abbreviations are used in this manuscript:

IBD	Inflammatory Bowel Diseases
CD	Crohn’s Disease
UC	Ulcerative Colitis
CDAI	Crohn’s Disease Activity Index
NHANES	National Health and Nutrition Examination Survey
CES-D	Center for Epidemiologic Studies Depression
HGS	Hand Grip Strength
IPAQ	International Physical Activity Questionnaires
MAMC	Mid-Arm Muscle Circumference
MUAC	Mid-Upper Arm Circumference
TSF	Triceps Skinfold Thickness
BMI	Body Mass Index
QoL	Quality of Life
SIBDQ	Short Inflammatory Bowel Disease Questionnaire
IQR	Interquartile Range
SD	Standard Deviation
OR	Odds Ratio
CI	Confidence Interval
TNF	Tumor Necrosis Factor
ICD	International Classification of Diseases
